# Isomorphism: ‘molecular similarity to crystal structure similarity’ in multicomponent forms of analgesic drugs tolfenamic and mefenamic acid

**DOI:** 10.1107/S205225251901604X

**Published:** 2020-01-07

**Authors:** Subham Ranjan, Ramesh Devarapalli, Sudeshna Kundu, Subhankar Saha, Shubham Deolka, Venu R. Vangala, C. Malla Reddy

**Affiliations:** a Department of Chemical Sciences, Indian Institute of Science Education and Research (IISER), Kolkata, Mohanpur Campus, Mohanpur 741 246, India; b Department of Pharmaceutical Sciences and Technology, Birla Institute of Technology, Mesra, Ranchi 835 215, India; c Centre for Pharmaceutical Engineering Science, School of Pharmacy and Medical Sciences, University of Bradford, Richmond Road, Bradford BD7 1DP, UK

**Keywords:** crystal engineering, polymorphism, co-crystals, isomorphism

## Abstract

The non-steroidal anti-inflammatory drugs mefenamic acid (MFA) and tolfenamic acid (TFA) have a close resemblance in their molecular scaffold, whereby a methyl group in MFA is substituted by a chloro group in TFA. The present study demonstrates the isomorphous nature of these compounds in a series of their multicomponent solids. Furthermore, the unique nature of MFA and TFA has been demonstrated while excavating their alternate solid forms in that, by varying the drug (MFA or TFA) to coformer [4-di­methyl­amino­pyridine (DMAP)] stoichiometric ratio, both drugs have produced three different types of multicomponent crystals, viz. salt (1:1; API to coformer ratio), salt hydrate (1:1:1) and cocrystal salt (2:1). Interestingly, as anticipated from the close similarity of TFA and MFA structures, these multicomponent solids have shown an isomorphous relation. A thorough characterization and structural investigation of the new multicomponent forms of MFA and TFA revealed their similarity in terms of space group and structural packing with isomorphic nature among the pairs. Herein, the experimental results are generalized in a broader perspective for predictably identifying any possible new forms of comparable compounds by mapping their crystal structure landscapes. The utility of such an approach is evident from the identification of polymorph VI of TFA from hetero-seeding with isomorphous MFA form I from acetone–methanol (1:1) solution. That aside, a pseudopolymorph of TFA with di­methyl­formamide (DMF) was obtained, which also has some structural similarity to that of the solvate MFA:DMF. These new isostructural pairs are discussed in the context of solid form screening using structural landscape similarity.

## Abstract 2

Many single- and multicomponent forms of two structurally related pharmaceutical drugs, tolfenamic acid and mefenamic acid, are isostructural in nature. This observation prompted the search for hidden forms by mapping the structural landscapes of the two drugs.

## 1. Introduction   

In crystal engineering, the aspects of isostructurality and polymorphism have always been intriguing for implications on the crystal packing and the potential impact on the properties of crystalline solids. Polymorphism is defined as the distinctive crystalline arrangements of a substance with the same chemical composition (Bernstein, 2002 ▸). On the other hand, two crystals are said to be isostructural if they have the same crystal structure but not necessarily the same cell dimensions nor the same chemical composition, whereas two crystalline solids are isomorphous if both have the same unit-cell dimensions and space group (Kálmán *et al.*, 1993 ▸). It is perceived that the polymorphs discovered are often seren­dipitous, difficult to control and cause disadvantages over benefits (Llinàs & Goodman, 2008 ▸). Polymorphs may display very diverse properties (Bauer *et al.*, 2001 ▸; Bag *et al.*, 2012 ▸; Krishna *et al.*, 2013 ▸; Saha & Desiraju, 2018*a* ▸).

On the other hand, isostructurality has advantages such as reliable structure versus property knowledge transfer (Chennuru *et al.*, 2017 ▸; Wood *et al.*, 2012 ▸; Reddy *et al.*, 2006 ▸; Krishna *et al.*, 2016 ▸; Saha & Desiraju, 2017*b* ▸), and formation of solid solutions for tuning physicochemical properties (Elgavi *et al.*, 1973 ▸; Vangala *et al.*, 2002 ▸; Pigge *et al.*, 2006 ▸). In general, polymorphism and isostructurality are perceived as opposite phenomena. Coles *et al.* recently demonstrated a counter-intuitive case of two polymorphic forms with close structural resemblance which they called *isostructural polymorphs* (Coles *et al.*, 2014 ▸; Fábián & Kálmán, 2004 ▸). This term has also been used in a different (rather unfitting) context to describe isostructural relationships between two forms of polymorphic analogous molecular pairs (Nath *et al.*, 2008 ▸).

Methyl–chloro (Me, 19 Å^3^; Cl, 21 Å^3^) exchange is a well examined topic in isostructural studies (Ebenezer *et al.*, 2011 ▸; Edwards *et al.*, 2001 ▸). Kitaigorodskii stated that this exchange depends on volume and shape considerations rather than electronic factors (Kitaigorodskii, 1973 ▸). However, this interchange rule is broken when directional and/or electronic interactions are involved in the crystal packing (Desiraju & Sarma, 1986 ▸; Edwards *et al.*, 2006 ▸). This means that, in some instances, the volume/shape considerations alone are inadequate and electronic factors must also be considered (SeethaLekshmi *et al.*, 2014 ▸; Braga *et al.*, 2009 ▸; Nath & Nangia, 2012 ▸; Reddy *et al.*, 2006 ▸). There have been attempts to achieve forms with Cl/Me interchange. Braga *et al.* achieved a new polymorph of *p*-methyl­benzyl alcohol, which is isomorphous with the crystal of *p*-chloro­benzyl alcohol, by hetero-seeding with a small quantity of the latter (Romasanta *et al.*, 2017 ▸). Although there have been several case studies on isostructurality consisting of single-component forms, only a few examples of multicomponent forms are reported in the literature (Cinčić *et al.*, 2008*a* ▸; Fandaruff *et al.*, 2015 ▸; Clarke *et al.*, 2012 ▸; Galcera & Molins, 2009 ▸).

In this study, we explored non-steroidal anti-inflammatory drugs tolfenamic (TFA) and mefenamic acids (MFA) to investigate the existence of isostructurality in their multicomponent solids. TFA and MFA are of interest in this study because, at the molecular level, there is a difference just in one position where the methyl group in MFA is replaced by the chloro group in TFA (Scheme 1). It has been reported that these two fenamates exist in several polymorphic forms owing to their conformational flexibility among the bridged amino group and carboxyl­ated phenyl group (SeethaLekshmi & Row, 2012 ▸; Bouanga Boudiombo & Jacobs, 2016 ▸; Wittering *et al.*, 2015 ▸). TFA and MFA are known to exist in several polymorphic forms. Interestingly, crystal packing analyses reveal that amongst these, only form V of TFA is isomorphous to that of form II of MFA despite their ability to show Cl/Me interchange. From an isomorphous crystal point of view, a question arises as to whether one is likely to find some new forms for MFA that are isomorphous with that of known polymorphic forms of TFA and vice versa. One of the primary goals of this study is to unravel such hidden or new polymorphs of these active pharmaceutical ingredients (APIs) to expand the crystal structure landscape. This study allowed us to identify a new polymorph (VI) of TFA by hetero-seeding with the crystals of polymorph I of MFA from solution methods (Ranjan *et al.*, 2017*b* ▸). During the same period, Price and coworkers carried out an exhaustive study involving computational (crystal structure prediction) and experimental techniques on the single-component polymorphic forms of fenamates and successfully showed the effective use of this method by obtaining several new forms using the known isomorphous forms as templates in the sublimation method (Case *et al.*, 2018 ▸).
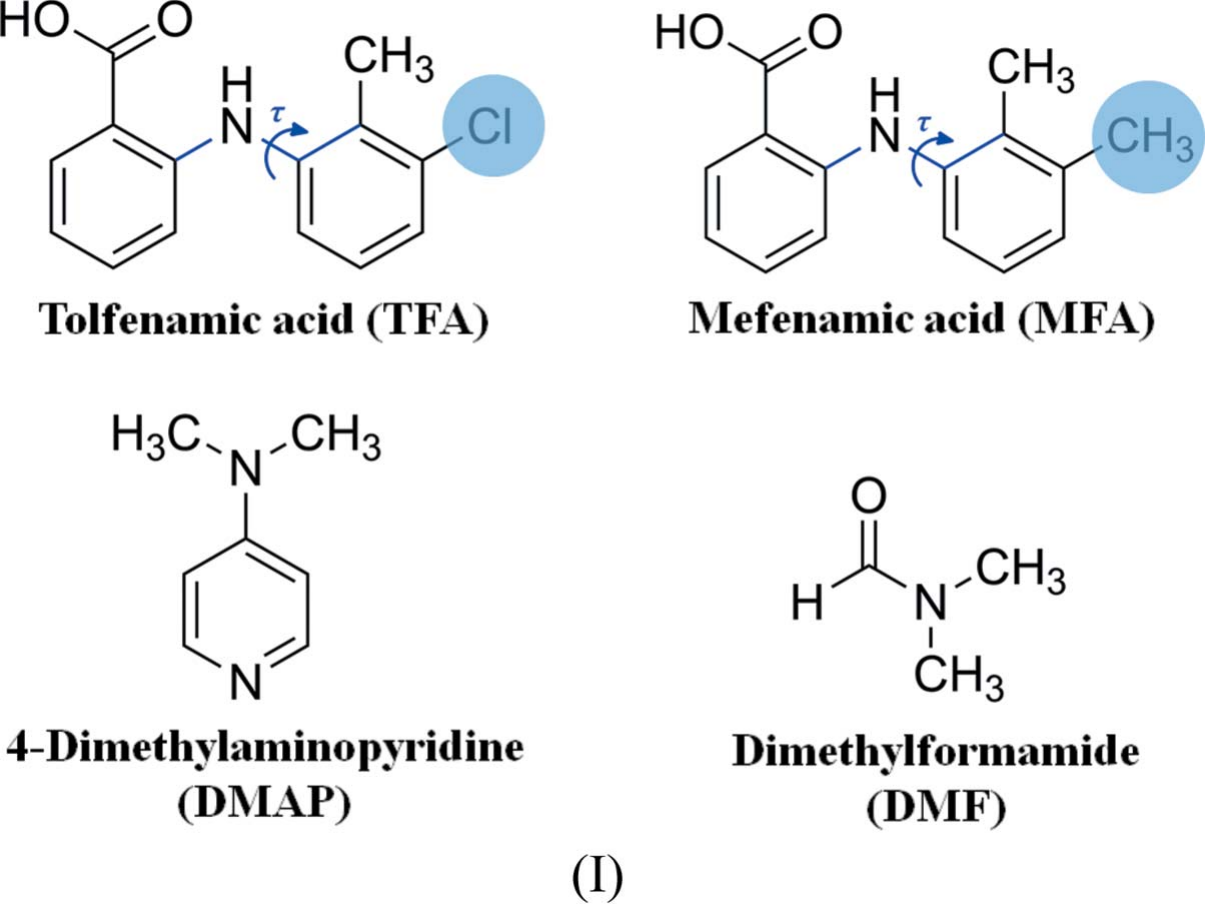


Among the documented literature, the following two reports are of interest in this study. Fábián *et al.* have investigated the 1:2 (API to coformer ratio) co-crystal formation of all the fenamates with nicotinamide (Fábián *et al.*, 2011 ▸; Utami *et al.*, 2016 ▸). Later, Surov *et al.* described the similarity in crystal packing of 2:1 co-crystals of tolfenamic acid and mefenamic acid with 4,4′-bi­pyridine (Surov *et al.*, 2015 ▸). However, in both cases, the authors have not explored the concept of isomorphism. The present work reports the structural studies of new multicomponent solids of TFA or MFA with 4-di­methyl­amino­pyridine (DMAP), which is known to form cocrystals/salts with APIs (Vangala *et al.*, 2013 ▸; Ranjan *et al.*, 2017*a* ▸) in varying stoichiometric ratios. The concepts of solid-form screening, isostructurality, polymorphism and similarity in structural landscapes of related compounds are discussed.

## 2. Experimental   

### 2.1. Materials   

TFA, MFA, DMAP, DMF and other solvents were purchased from Sigma–Aldrich and were used without further purification.

### 2.2. Preparation of solid forms   

The commercially available parent drugs TFA and MFA were separately ground with DMAP in different molar ratios, 1:1, 1:2 and 2:1 (drug to coformer ratio), with a few drops of methanol, which resulted in the 1:1 salt, 1:1:1 salt monohydrate and 2:1 co-crystal salt, respectively. The amounts of TFA and DMAP used in the synthesis of the salt, salt-monohydrate and cocrystal-salt were 50 and 23 mg, 50 and 46 mg, and 100 and 23 mg, respectively. Similarly, for the MFA–DMAP salt, salt monohydrate and cocrystal-salt, the amounts of MFA and DMAP used were 50 and 15 mg, 50 and 30 mg, and 100 and 15 mg, respectively. Single crystals were prepared by dissolving the respective ground powders in 10 ml acetone or an acetone–methanol solvent mixture (5 ml each) under ambient conditions and left undisturbed for 3 to 5 days. To get TFA–DMF in the solvated form, 50 mg of TFA was dissolved in 10 ml DMF under ambient conditions and was left undisturbed for 6–8 days. For TFA form VI preparation, 50 mg of TFA was dissolved in 10 ml of acetone:methanol (1:1) solvent mixture at 50 to 55°C to produce clear solution which was then allowed to cool without stirring. After cooling to 30°C, a couple of single crystals of MFA form I were added to the solution. Upon evaporation of the solvent, crystals of TFA form VI were obtained.

### 2.3. Single-crystal X-ray diffraction   

Single-crystal X-ray diffraction (SCXRD) data were recorded on a SuperNova Eos diffractometer using monochromatic Mo *K*α radiation (λ = 0.71073 Å) or Cu *K*α radiation (λ = 1.54184 Å) at room temperature (293 K) or low temperature (100 K). Using *Olex2* (Dolomanov *et al.*, 2009 ▸), the structures were solved with *SHELXT* (Sheldrick, 2015*a* ▸) using an intrinsic phasing algorithm and refined using *SHELXL* (Sheldrick, 2008 ▸, 2015*b* ▸). Atomic displacement parameters (ADPs) were refined for all non-hydrogen atoms. The hydrogens attached to the carbons were placed in calculated positions with fixed geometries using the riding model with isotropic ADPs constrained, *U*_iso_ = 1.5 *U*_eq_[C(*sp* ^3^)] and *U*_iso_ = 1.2 *U*_eq_[C(*sp* ^2^)]. Hydrogens of the N—H and O—H groups were located using difference Fourier maps and refined with distance restraints *d*_N–H_ = 0.87 ± 0.02 Å, *d*_O–H_ = 0.82 ± 0.02 Å, and isotropic ADPs constraints *U*_iso_ = 1.2 *U*_eq_(N), *U*_iso_ = 1.5 *U*_eq_(O). The crystal packing diagrams were prepared using *Mercury* (version 3.8).

### 2.4. Powder X-ray diffraction   

The bulk samples of all starting materials and new solid forms were characterized by powder X-ray diffraction (PXRD) analysis on a RigakuSmartLab using Cu *K*α radiation (1.54056 Å). For all experiments, the tube voltage and amperage were set at 40 kV and 35 mA, respectively. Each sample was scanned between 5 and 50° 2θ with a step size of 0.02°.

### 2.5. Differential scanning calorimetry   

Differential scanning calorimetry (DSC) was performed using a Mettler Toledo DSI1 *STAR* ^e^ instrument with ∼5 mg of samples crimped in hermetic aluminium crucibles (40 ml) by ramping from 50 to 250°C at a heating rate of 10°C min^−1^ under a dry nitro­gen atmosphere (flow rate 80 ml min^−1^). The data were analysed using *STAR* ^e^ software.

### 2.6. Thermogravimetric analysis   

Thermogravimetric analysis (TGA) was carried out using a Perkin Elmer, Diamond TG/DTA analyser, operated under a nitro­gen atmosphere with a heating rate of 10°C min^−1^ in the range 30**−**300°C.

## 3. Results and discussion   

### 3.1. Powder X-ray diffraction analysis   

Formation of a new solid phase and its purity were confirmed by PXRD analysis by matching the patterns with corresponding starting materials (see Figs. S13 and S14 of the supporting information). There is a good match between experimental and simulated PXRD patterns for all the multicomponent solids, except for two monohydrate salts. On the other hand, the mismatch between experimental and simulated PXRD patterns is not uncommon for hydrates or solvates (Clarke *et al.*, 2010 ▸). The preferable orientation and different data collection temperatures for the experimental powder pattern compared with that of the simulated pattern, which was generated using SCXRD data, could have contributed to this difference. On the other hand, we have seen the close match between the PXRD patterns of the same molar ratio pairs of multicomponent solids of TFA and MFA (*e.g*. 1:1 salts of TFA and MFA).

### 3.2. Crystal structure analysis   

#### 3.2.1. Multicomponent isomorphic crystals of analogous fenamic acids   

Cocrystallization of DMAP with fenamic acids is expected to yield a salt because of the ∼6 units of Δp*K*_a_ between DMAP and the corresponding API (TFA and MFA, p*K*_a_ = 3.7; DMAP, p*K*_a_ = 9.7). Furthermore, it is well documented that the salt form of an API can significantly change its physicochemical properties such as crystallinity, solubility and stability and hence can be considered as an alternative route for drug delivery (Maddileti *et al.*, 2014 ▸; Goud *et al.*, 2013 ▸).

Notably, all the solids including parent APIs and the new multicomponent forms crystallized in the triclinic 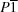 space group (Tables S1 and S2 of the supporting information). The 1:1 salt crystal structure possesses one molecule of TFA (or MFA) and DMAP each in the asymmetric unit, whereas the 1:1:1 salt monohydrate crystal structure (Ranjan *et al.*, 2017*b* ▸; Nechipadappu & Trivedi, 2017 ▸) contains a water molecule along with one TFA molecule (or MFA) and one DMAP molecule. Aside from this, the 2:1 co-crystal salt possesses two molecules of TFA (or MFA) and one molecule of DMAP in the asymmetric unit. The *ORTEP* representations of all the new solids are shown in Figs. S4–S11. Though the commercially available APIs TFA and MFA are not isomorphous (only isostructural), it is intriguing to perceive the isomorphic attribute in their multicomponent solids as their unit-cell parameters are near identical (Table 1 ▸). For further clarity, the isostructurality was quantitatively deciphered from the commonly used method which uses the unit-cell parameters of two crystal structures to calculate the unit-cell similarity index (Π) (Wood *et al.*, 2012 ▸; Sarmah *et al.*, 2017 ▸). If the compared structures are isostructural to a great extent, then Π should be close to zero, and if Π = 0, then they are isomorphous (Oliveira *et al.*, 2008 ▸). In the present case of the multicomponent pairs of TFA and MFA, the Π value is zero (up to the first decimal place, see Table 1 ▸), confirming the isomorphous nature of the pairs. Furthermore, PXRD similarity index scores for each pair and the RMSDs (root-mean-square deviations) were calculated from the packing similarity overlay with 20 molecules by allowing molecular differences and keeping default tolerance values. PXRD similarity and RMSD values were derived from the program *Mercury* (CSD version 3.8). The quantitative numbers are given in Table 1 ▸. The results obtained from crystal packing similarity calculations showed that 20 out of 20 molecules were matched in the pairs of 1:1 salts and 1:1:1 salt monohydrates, whereas in case of the 2:1 co-crystal salt pairs, only 9 out of 20 molecules were matched despite the Π value being close to zero. The reason for the match of such fewer molecules could be due to the additional disorder of the symmetrically-independent molecules in the respective crystal structures. Therefore, the comparable PXRD similarity scores in all three cases suggest that these pairs possess identical intermolecular interactions and lead to the same crystal packing (Fig. 2). Furthermore, we have utilized the ‘Xpac’ analysis to quantitatively measure the packing similarity between the TFA and MFA multicomponent series (Gelbrich & Hursthouse, 2006 ▸; Gelbrich *et al.*, 2012 ▸). This analysis provides the dissimilarity index (*x*) value, which is a measure of the deviation of two structures from perfect geometrical similarity (Fábián & Kálmán, 1999 ▸). In the current study, the ‘*x*’ values of the multicomponent solids of TFA and MFA are found to be 3.7 for 1:1 salts (Fig. S1), 1.0 for 1:1:1 salt monohydrates (Fig. S2) and 2.5 for 2:1 co-crystal salts (Fig. S3). Therefore, the ‘*x*’ values of the corresponding multicomponent solid pairs of TFA and MFA signify that all pairs are isomorphous.

In the structures of TFA and MFA multicomponent solids, some significant differences in the conformations were observed (see Table S3 for specifics). Although the torsion angles of the multicomponent solids deviate significantly from their parent API torsion angles, the torsion or dihedral angles among multicomponent solids for the same composition (*e.g.* 1:1 salts of TFA and MFA) are closely corroborated. In turn, the angles between the acid-holding aromatic ring of the API and DMAP pyridyl ring (θ) in the isomorphic pairs are quite close, which led to their identical close packing with nearly the same pattern of intermolecular interactions. As a result of such high twisting of rings, TFA and MFA molecules often show disorder in their structures.

**Figure 1:**
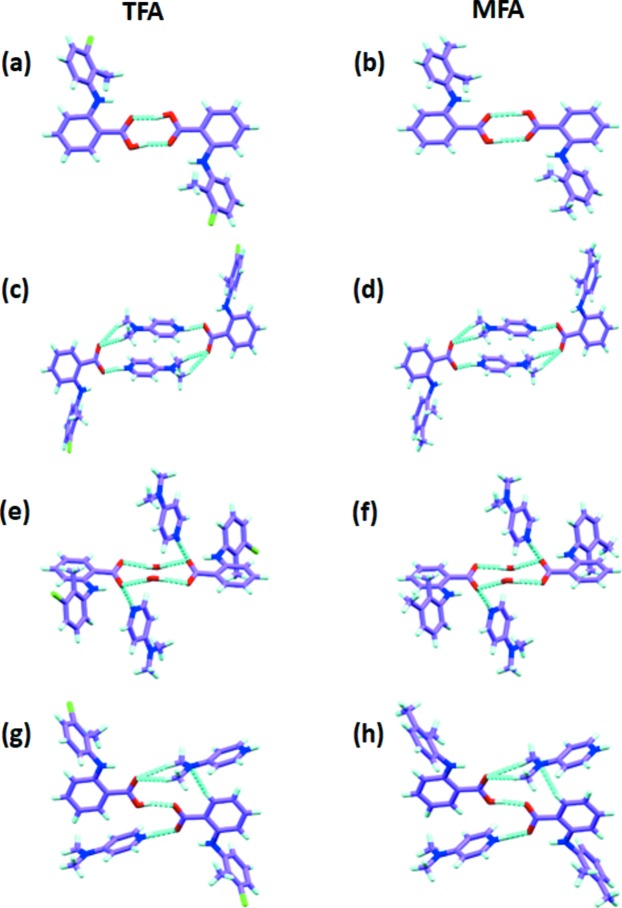
Synthons observed in crystal structures of parent APIs and multicomponent solids. (*a*) TFA form-I and (*b*) MFA form-I dimers (Andersen *et al.*, 1989 ▸; Lee *et al.*, 2006 ▸); (*c*) 1:1 TFA–DMAP and (*d*) MFA–DMAP salts; (*e*) 1:1:1 TFA–DMAP–H_2_O and (*f*) MFA–DMAP–H_2_O salt monohydrates; (*g*) 2:1 ratio of TFA–DMAP and (*h*) MFA–DMAP co-crystal salts.

In the crystal packing, both the parent APIs, TFA and MFA, form dimers and are close packed by several C—H⋯O and C—H⋯π interactions. However, in the new multicomponent solid forms of both APIs, the dimerization was disrupted either by a DMAP molecule (in the cases of 1:1 and 2:1) or a water molecule (in the case of 1:1:1). This is due to proton transfer from the acid (of API) to the DMAP molecule in all cases (Fig. 1 ▸). Furthermore, there were no noticeable Cl⋯Cl interactions observed in the parent TFA as well as in its multicomponent solids. If the Cl⋯Cl interactions have no effect on the packing of TFA crystals, then the methyl-substituted isostructural compound MFA would also display similar packing (Landenberger *et al.*, 2013 ▸). As a result, we could expect the isostructural/isomorphous packing in both TFA and MFA multicomponent solids of the Cl and CH_3_ groups to behave in a similar fashion in crystal packing for their comparable volumes, 19 and 24 Å^3^, respectively (Desiraju & Sarma, 1986 ▸).

**Figure 2:**
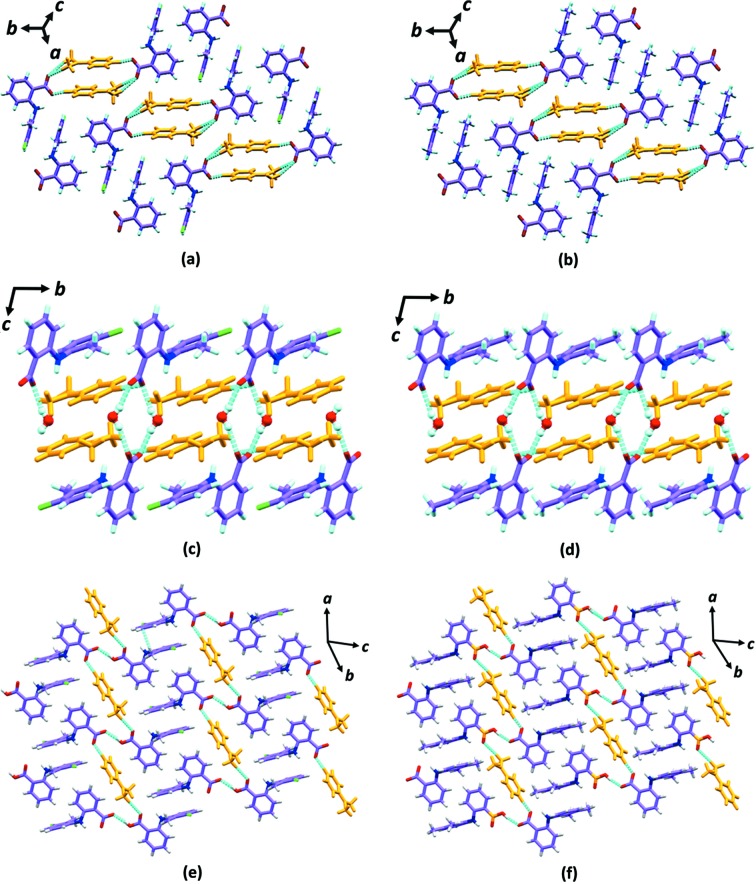
Crystal packing in (*a*) 1:1 TFA–DMAP and (*b*) MFA–DMAP salts; (*c*) 1:1:1 TFA–DMAP–H_2_O and (*d*) MFA–DMAP–H_2_O salt monohydrates; (*e*) 2:1 TFA-DMAP and (*f*) MFA–DMAP co-crystal salts, respectively. For clarity, the pyridine molecules are shown in orange and C—H⋯π interactions have been omitted.

In the 1:1 salt crystal packing, two molecules of TFA (or MFA) and two molecules of DMAP form a tetrameric synthon *via* N—H⋯O and C—H⋯O hydrogen bonds [Figs. 2 ▸(*a*) and 2 ▸(*b*)]. The adjacent tetrameric synthons are joined by moderately strong C—H⋯O interactions along the *b* direction. The motifs are further stacked by antiparallel π-stacking interactions between chlorinated (or methyl­ated in MFA) phenyl rings of TFA along [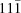]. Whereas in the case of the 1:1:1 salt hydrate, two molecules of TFA (or MFA) and two molecules of water form the tetrameric synthon with strong O—H⋯O hydrogen bonds [Figs. 1 ▸(*e*) and 1 ▸(*f*)]. These tetramers grow along the *c* direction in a 1D chain by edge-on (or T-shaped) C—H⋯π interactions between adjacent TFA (or MFA) molecules of the tetrameric synthon. Along the other two crystallographic directions *a* and *b*, the 1D chains are joined by DMAP molecules *via* strong N—H⋯O and weak C—H⋯O interactions [Figs. 2 ▸(*c*) and 2 ▸(*d*)]. In the third case, the 2:1 co-crystal salt, the alternative TFA (or MFA) and DMAP molecules form an infinite 1D chain (which resembles an open catemer) *via* strong O—H⋯O, N—H⋯O and C—H⋯O interactions. Furthermore, these 1D chains are interlocked by π-stacking interactions of TFA chlorinated (or MFA methyl­ated) phenyl rings along (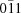) as shown in Figs. 2 ▸(*e*) and 2 ▸(*f*). The geometrical parameters of hydrogen bonding in all the complexes are given in the Table S4.

#### 3.2.2. Polymorphism among analogous fenamic acids   

Although our initial aim was to find the existence of isomorphism in multicomponent solids, we serendipitously discovered a new polymorph of TFA while co-crystallizing with DMAP, hereafter designated as TFA form VI (Ranjan *et al.*, 2017*b* ▸). This form was obtained by Price and co-workers in their study (Case *et al.*, 2018 ▸). SCXRD data of this polymorph revealed that its cell parameters are identical to form I of MFA with unit-cell similarity index Π = 0.015. In addition, the high similarity index of PXRD (0.953) and low RMSD (0.132 Å) values suggest close structural similarity (see Table 2 ▸). Our attempts to reproduce form VI by the same co-crystallization procedure were unsuccessful. Even the co-crystallization attempts in different stoichiometric ratios or with different reaction conditions were also unsuccessful. However, interestingly, form VI could be obtained by the hetero-seeding crystallization method (Braga *et al.*, 2009 ▸; Ebenezer *et al.*, 2011 ▸) from its analogue isostructural form: form I of MFA from a 1:1 mixture of acetone and methanol solution by slow evaporation.

**Figure 3:**
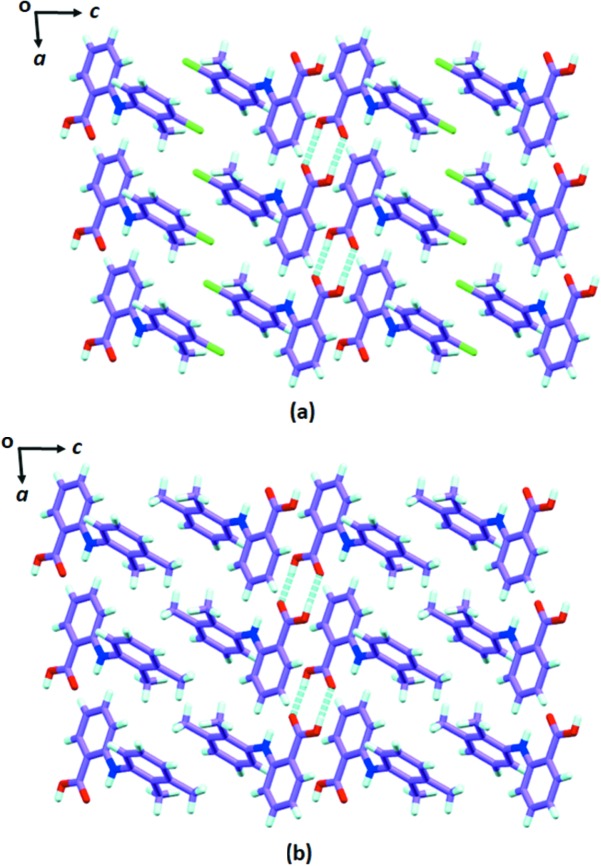
Crystal packing of (*a*) TFA form-VI and (*b*) MFA-form-I in the *ac* plane.

Nevertheless, the crystal packing of TFA form VI, which is isomorphous to MFA form I (McConnell, 1976 ▸), crystallizes in the triclinic space group 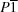 with one molecule in the asymmetric unit. The TFA molecules form dimers. The alternate dimers along the *b* axis are held together by edge-to-face C—H⋯π (2.78 Å) interactions. Along the *c* direction, these dimer stacks are arranged in an anti-parallel fashion *via* C—H⋯π (2.71 Å) interactions. Therefore, the crystal structure view in the *ac* plane resembles the zipper-type interlocked packing of dimers [Fig. 3 ▸(*a*)]. The structural similarity between these isomorphous forms (MFA form I and TFA form VI) can be seen in Fig. 3 ▸.

#### 3.2.3. Non-isomorphous pseudopolymorphs among analogous fenamic acids   

**Figure 4:**
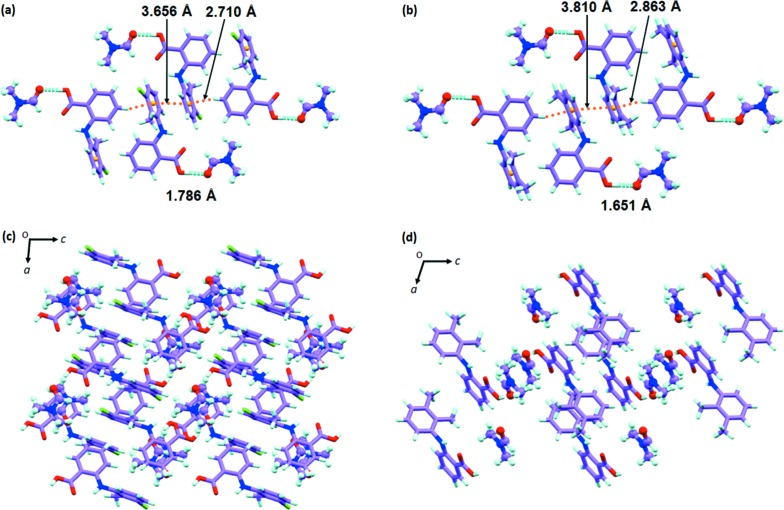
Dimers in (*a*) TFA–DMF and (*b*) MFA–DMF, which interact *via* π⋯π and C—H⋯π interactions, show their structural similarity at the local level. Comparison of crystal packing of (*c*) TFA–DMF and (*d*) MFA–DMF solvates highlights differences when viewed from other directions. The DMF molecules are shown in ball and stick form for easy visualization of its interactions with fenamates.

To further demonstrate the viability of this concept, we attempted to obtain the other unknown forms for both fenamates. Consequently, we were able to obtain the new pseudopolymorph TFA–DMF solvate by crystallizing the TFA in DMF solvent (SeethaLekshmi & Row, 2012 ▸). However, the SCXRD data of this new solvate revealed that its cell parameters do not exactly match with its analogue, the MFA–DMF solvate. The unit-cell similarity index of this pair (∼0.149) is not close to zero and only 15 out of 20 molecules could be matched while calculating the packing similarity (Table 3 ▸). The deviation is caused by changes in the structural packing. Unlike MFA–DMF, which consists of one molecule each of MFA and DMF in the asymmetric unit, the TFA–DMF has two TFA and two DMF molecules in the asymmetric unit. As a result, the volume is roughly doubled to that of MFA–DMF. In both these pseudopolymorphs, the DMF molecule interrupts the acid dimer and forms TFA–DMF or MFA–DMF dimers through O—H⋯O and C—H⋯O hydrogen bonds. As shown in Figs. 4 ▸(*a*) and 4 ▸(*b*), the pseudopolymorphs have the same interactions (π⋯π and C—H⋯π) between the dimers, hence they have some structural similarity; however, these are non-isostructural. The difference in dihedral angle (∼9°, see Fig. S12) between symmetrically independent TFA molecules in TFA–DMF leads to significant changes in close packing compared with MFA–DMF [Figs. 4 ▸(*c*) and 4 ▸(*d*)], which in turn makes them non-isostructural or non-isomorphous pairs.

### 3.3. Thermal analysis   

Thermal behaviour of all the multicomponent solids and polymorphs was investigated by DSC and TGA, and the profiles are presented in Figs. S15 and S16. The melting points of TFA, MFA and DMAP are in the ranges 206.8–215.2, 230–231 and 110–113°C, respectively. All of the multicomponent solid forms exhibit distinct melting points to that of their respective starting materials, which is corroborated by PXRD and SCXRD results, suggesting the formation of new crystalline phases. The melting points of the TFA–DMAP (1:1) salt, the TFA–DMAP (2:1) co-crystal salt, the MFA–DMAP (1:1) salt and the MFA–DMAP (2:1) co-crystal salt were observed at 158, 178, 165 and 162°C, respectively. Negligible weight losses were observed in their respective TGA profiles, thus confirming that the endothermic peak is its melting temperature. The endothermic melting peaks of TFA–DMAP–H_2_O (1:1:1) and MFA–DMAP–H_2_O (1:1:1) were observed at 152 and 162°C, respectively. The TGA profiles of these solids established the stoichiometry of water in these hydrates (Du *et al.*, 2009 ▸). Weight losses of 4.45 and 4.9% were observed in the temperature ranges 72–98 and 78–97°C in the TGA trace of TFA–DMAP–H_2_O (1:1:1) and MFA–DMAP–H_2_O (1:1:1), respectively, which correspond to the loss of one water molecule. This value is in accordance with the theoretical mass losses of 4.7 and 4.48% for desolvation of one mole of water from the respective crystal lattices and matching well with the calculated amount of water in the crystal structure. After the loss of water, TFA–DMAP–H_2_O (1:1:1) was stable up to 142°C and then began to decompose upon further heating. Similarly, MFA–DMAP–H_2_O (1:1:1) was stable up to 157°C.

Notably, the isomorphous pairs showed different melting points (physical properties) despite their similar structural packing. These results suggest that the properties of isostructural pairs can be directly correlated to chemical contribution from constituents, as mentioned by Jones and co-workers (Cinčić *et al.*, 2008*b* ▸).

We have also performed DSC experiments for TFA form VI and the TFA–DMF solvate and noticed melting endotherms at 221 and 209°C, respectively. In addition, we observed an additional endotherm that coud be ascribed to the solvent molecule in TFA–DMF. The observed weight loss of 17.6% in the temperature range 55–97°C for TFA–DMF corresponds to solvent evaporation. The remaining solids have shown no weight loss before their melting points, which confirms their purity and stability.

## 4. Hirshfeld surface analysis   

**Figure 5:**
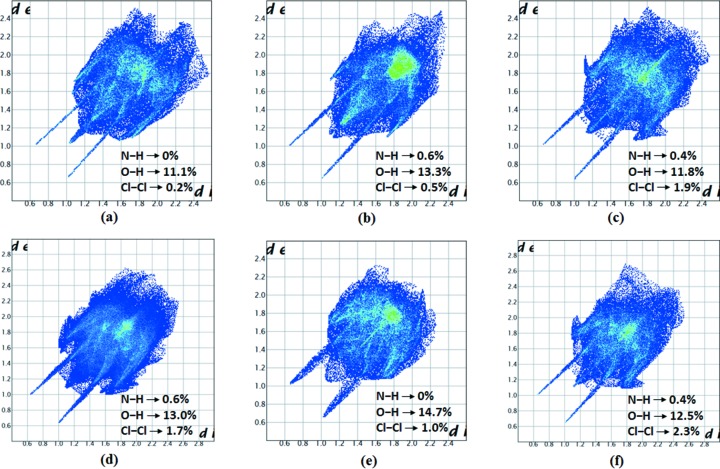
The fingerprint plots of polymorphs of TFA in ascending order from form I to form VI.

Because it is difficult to quantify the short contacts present in a structure from either *Mercury* or other useful crystallography software, we used Hirshfeld surface analysis. Moreover, it is easy to plot and compare the interactions present in different polymorphic forms of a substance. Here, the newly obtained TFA polymorph VI is compared with the other five known polymorphs by plotting fingerprint plots (Fig. 5 ▸). The common interactions that participate in hydrogen bonding: O⋯H, N⋯H and other non-hydrogen bonding interactions are listed in the respective plots. From the overall interaction contribution, it is clear that the interactions in the new form are also in line with the reported polymorphs.

## 5. Discussion   

### 5.1. Isostructurality and the relationship with crystal structural landscape   

The investigation of crystal structural landscapes helps in understanding various dynamic events that occur during crystallization, including polymorphs, pseudopolymorphs and high-*Z*′ issues (Mukherjee *et al.*, 2011 ▸). In principle, for a given molecule there exists a large number of virtual crystal structures within a narrow energy window. Many factors control a particular crystal structure formation. Accessing different structure types or, in other words, data points in a crystal structure landscape for a given system is a difficult task. This can be done experimentally (Chakraborty *et al.*, 2018 ▸) or computationally through crystal structure prediction (CSP) (Thakur *et al.*, 2015 ▸). However, CSP does not provide the information on the final experimental outcome under given crystallization conditions as it does not consider the kinetic issues associated with a crystallization event. By exploring the crystal structure landscape, one may find means to achieve a particular crystal structure with new synthons that are not readily accessible (Saha & Desiraju, 2018*b* ▸). Obtaining such structures would be more useful when a specific structure type can only display a particular property (Saha & Desiraju, 2017*a* ▸). Synthons encapsulate kinetic information regarding the process of crystallization. According to classical nucleation theory, synthons can be generated in solution and finally transfer into the crystal structure (Parveen *et al.*, 2005 ▸; Davey *et al.*, 2006 ▸). Understanding these events helps in structural profiling which, in turn, can guide us to develop crystal engineering strategies.

**Figure 6:**
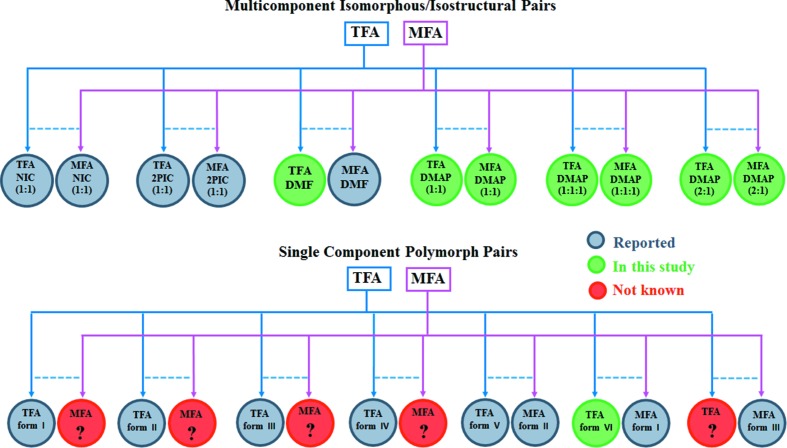
Isomorphous/isostructural pairs of multicomponent (top) and single-component (bottom) solids of TFA and MFA.

The present work considers experimental exploration of crystal structure landscapes for stoichiometric multicomponent fenamic acid drugs TFA and MFA. The only substitutional change is Cl/Me exchange on the basic fenamic acid molecular scaffold, *i.e.* the molecular similarity between TFA and MFA makes them ideal candidates to compare their individual crystal structural landscapes as a whole. In this regard, the multicomponent solids of TFA and MFA with DMAP and/or DMF are considered. A total of six binary structures for TFA were analysed in that three are different forms with DMAP. Analyses of crystal structures for MFA reveal the existence of identical or very similar crystal structures for each TFA type. Such a high degree of matching suggests that, by exploring the landscape for a model system, one can practically find possible crystal structures for other similar systems (Chennuru *et al.*, 2017 ▸). Similarly, crystal structures of single-component TFA and MFA are analysed. We analyzed the six polymorphic forms of TFA (I–VI) with the three MFA forms (I–III) known thus far. However, crystal structures were matched for barely two pairs (TFA form V and MFA II; TFA form VI and MFA form I) (Fig. 6 ▸). Accordingly, one can assume that crystal structure pattern types for five pairs, which are placed in different energy data points in the landscape diagram, are known or experimentally accessed. It should also be mentioned that not only are the structural patterns of these polymorphs different, but also the major synthons differ significantly, indicating the possible influence of both geometrical and chemical factors. Such observation is uncommon (Dubey *et al.*, 2014 ▸; Saha & Desiraju, 2018*b* ▸). Here we see the existence of dimers and catemers for the same coformer DMAP. Dimers with multipoint recognition are generally known to be thermodynamically favoured, whereas single-point catemers are kinetically preferred. Such differences in structural patterns may lead to changes in physicochemical properties, such as solubility, stability, optical activity and nonlinear optical behaviour.

## 6. Conclusions   

Existence of isomorphism in the multicomponent solids of TFA and MFA has been thoroughly investigated by considering the similarity of their crystal structure landscapes. The quantitative numbers of unit-cell similarity, PXRD similarity index and RMSD values suggest that the multicomponent crystals of TFA and MFA with DMAP and/or DMF in the same molar ratio are structurally very closely associated. In addition, the discovery of a new polymorph of TFA and a pseudopolymorph TFA–DMF solvate is in line with the concept of isomorphism. Hence, this study demonstrates that isomorphism will be a promising guiding principle for using crystal structure landscape similarity to uncover hidden unknown structures of closely resembling compounds, *i.e.* molecular similarities. Further work is ongoing in our laboratory to unravel the other hidden single-component forms of TFA and MFA.

It may be possible to use one model molecular system to explore the crystal structure landscape for other systems with molecular similarity (Chakraborty *et al.*, 2018 ▸; Case *et al.*, 2018 ▸). Such an approach can help to predict or compare properties of different structure types of analogous compounds in comparison with the model system (Krishna *et al.*, 2016 ▸), without exploring each structure for individual compounds. If different forms are known for the model system, then measurements of their properties will reveal the suitability of targeted properties or applications (Saha *et al.*, 2018 ▸). One can then target that particular structure type in an analogus compound to improve the property further (for example, by functional-group exchange) by using the knowledge of the isostructural behaviour of molecules.

## Supplementary Material

Crystal structure: contains datablock(s) tfa_dmap_1-1, tfa-dmap-H2O_1-1-1, tfa-dmap_2-1, tfa_form-vi, tfa_dmf, mfa-dmap_1-1, mfa-dmap-H2O_1-1-1, mfa-dmap_2-1. DOI: 10.1107/S205225251901604X/ed5019sup1.cif

Structure factors: contains datablock(s) tfa_dmap_1-1. DOI: 10.1107/S205225251901604X/ed5019tfa_dmap_1-1sup2.hkl

Structure factors: contains datablock(s) tfa-dmap-H2O_1-1-1. DOI: 10.1107/S205225251901604X/ed5019tfa-dmap-H2O_1-1-1sup3.hkl

Structure factors: contains datablock(s) tfa-dmap_2-1. DOI: 10.1107/S205225251901604X/ed5019tfa-dmap_2-1sup4.hkl

Structure factors: contains datablock(s) tfa_form-vi. DOI: 10.1107/S205225251901604X/ed5019tfa_form-visup5.hkl

Structure factors: contains datablock(s) tfa_dmf. DOI: 10.1107/S205225251901604X/ed5019tfa_dmfsup6.hkl

Structure factors: contains datablock(s) mfa-dmap_1-1. DOI: 10.1107/S205225251901604X/ed5019mfa-dmap_1-1sup7.hkl

Structure factors: contains datablock(s) mfa-dmap-H2O_1-1-1. DOI: 10.1107/S205225251901604X/ed5019mfa-dmap-H2O_1-1-1sup8.hkl

Structure factors: contains datablock(s) mfa-dmap_2-1. DOI: 10.1107/S205225251901604X/ed5019mfa-dmap_2-1sup9.hkl

Supporting information. DOI: 10.1107/S205225251901604X/ed5019sup10.pdf

CCDC references: 1532470, 1532471, 1532472, 1532473, 1532474, 1532475, 1532476, 1532477

## Figures and Tables

**Table 1:** Unit-cell parameters, root-mean-square deviation (RMSD), PXRD similarity and unit-cell similarity index (Π) for TFA and MFA multicomponent solids The unit-cell parameters with estimated standard deviations are provided in the supporting information, see Tables S1 and S2.

	Unit-cell parameters (Å, °, Å^3^)	RMSD (Å)	PXRD similarity	Unit-cell similarity index
TFA–DMAP (1:1)	*a* = 7.9299, *b* = 9.3219, *c* = 13.5862	0.219	0.987	0.010
	α = 87.768, β = 76.928, γ = 76.025			
	*V* = 949.20			
MFA–DMAP (1:1)	*a* = 7.7575, *b* = 9.4727, *c* = 13.3076			
	α = 87.515, β = 78.596, γ = 74.174			
	*V* = 922.20			
TFA–DMAP–H_2_O (1:1:1)	*a* = 7.7631, *b* = 8.0250, *c* = 16.2297	0.088	0.989	0.001
	α = 101.784, β = 98.374, γ = 90.687			
	*V* = 978.33			
MFA–DMAP–H_2_O (1:1:1)	*a* = 7.7248, *b* = 8.0592, *c* = 16.2531			
	α = 101.711, β = 98.743, γ = 90.160			
	*V* = 978.68			
TFA–DMAP (2:1)	*a* = 10.8864, *b* = 12.2705, *c* = 13.7811	0.872	0.982	0.011
	α = 106.966, β = 105.782, γ = 103.324			
	*V* = 1595.92			
MFA–DMAP (2:1)	*a* = 10.7678, *b* = 11.9673, *c* = 13.7860			
	α = 106.151, β = 105.854, γ = 103.490			
	*V* = 1546.4			

**Table 2:** Unit-cell parameters, root-mean-square deviatiom (RMSD), PXRD similarity and unit-cell similarity index (Π) for TFA and MFA polymorphs

	Unit-cell parameters (Å, °, Å^3^)		RMSD (Å)	PXRD similarity	Unit-cell similarity index
TFA form-VI	*a* = 6.7049, *b* = 7.2778, *c* = 14.1630		0.132	0.953	0.015
(New form)	α = 77.167, β = 79.908, *γ =* 65.487				
	*V* = 610.42				
MFA form-I	*a* = 6.8144, *b* = 7.3256, *c* = 14.4196				
(McConnell, 1976 ▸)	α = 76.648, β = 79.178, *γ =* 65.547				
	*V* = 634.08				
TFA form-V	*a* = 7.6488, *b* = 9.0160, *c* = 9.4184				
(López-Mejías *et al.*, 2009 ▸)	α = 107.385, β = 92.062, γ = 101.662		Could not be done due to heavy disorder of the molecules.		0.005
	*V* = 603.806				
MFA form-II	*a* = 7.70630, *b* = 9.10160, *c* = 9.39700				
(Lee *et al.*, 2006 ▸)	α = 107.2850, β = 91.4080, γ = 101.8040				
	*V* = 613.454				

**Table 3:** Unit-cell parameters, root-mean-square deviation (RMSD), PXRD similarity and unit-cell similarity index (Π) for TFA and MFA solvates

	Unit-cell parameters (Å, °, Å^3^)		RMSD (Å)	PXRD similarity	Unit-cell similarity index
TFA–DMF	*a* = 10.4803, *b* = 11.8423, *c* = 13.3309		0.229	0.948	0.149
	α = 94.335, β = 95.884, *γ =* 102.871				
	*V* = 1596.16				
MFA–DMF	*a* = 7.4730, *b* = 9.559, *c* = 13.306				
	α = 105.070, β = 103.780, γ = 103.410				
	*V* = 846.512				
