# Supplementary material for: Isomorphism: ‘molecular similarity to crystal structure similarity’ in multicomponent forms of analgesic drugs tolfenamic and mefenamic acid
Source: IUCrJ. 2020 Jan 7;7(Pt 2):173–83. doi: 10.1107/S205225251901604X (PMC7055380; doi:10.1107/S205225251901604X)
Supplement: Supplementary file 10 [file m-07-00173-sup10.pdf]

Electronic Supporting Information

Isomorphism: 'Molecular Similarity to Crystal Structure Similarity' in multicomponent forms of Analgesic Drugs, Tolfenamic and Mefenamic Acids

Subham Ranjan,[a] Ramesh Devarapalli,[a] Sudeshna Kundu,[b] Subhankar Saha,[a] Shubham Deolka,[a] Venu R. Vangala[c]* and C. Malla Reddy[a]*

[a]*Department of Chemical Sciences, Indian Institute of Science Education and Research (IISER) Kolkata, Mohanpur Campus, Mohanpur 741 246, India*
[b]*Department of Pharmaceutical Sciences and Technology, Birla Institute of Technology, Mesra, Ranchi, 835 215, India*
[c]*Centre for Pharmaceutical Engineering Science, School of Pharmacy and Medical Sciences, University of Bradford, Richmond Road, Bradford, BD7 1DP, United Kingdom*

(15 pages)

Table of Contents

Table/ Figure	Contents	Page No
**Table S1**	Crystallographic data and structure refinement parameters of TFA multicomponent solids and a polymorph (TFA form-VI).	S3, S4
**Table S2**	Crystallographic data and structure refinement parameters of MFA multicomponent solids.	S4, S5
**Table S3**	Selected torsion angles, dihedral angles and the angle between the acid containing aromatic ring of fenamate and the DMAP pyridyl ring.	S6
**Table S4**	Geometrical parameters of hydrogen bonds observed in the crystal structure.	S6,S7
**Fig. S1**	3D supramolecular constructs between MFA-DMAP (1:1) and TFA-DMAP (1:1) deduced from XPac analysis.	S8
**Fig. S2**	3D supramolecular constructs between MFA-DMAP-H_2O (1:1:1) and TFA-DMAP-H_2O (1:1:1) deduced from XPac analysis.	S8
**Fig. S3**	3D supramolecular constructs between MFA-DMAP (2:1) and TFA-DMAP (2:1) deduced from XPac analysis.	S8
**Fig. S4**	ORTEP representation of TFA-DMAP (1:1).	S9
**Fig. S5**	ORTEP view of TFA-DMAP-H_2O (1:1:1).	S9
**Fig. S6**	ORTEP representationof the TFA-DMAP (2:1).	S10
**Fig. S7**	ORTEP representation of TFA form-VI.	S10
**Fig. S8**	ORTEP view of TFA-DMF.	S10
**Fig. S9**	ORTEP representation of MFA-DMAP (1:1).	S11
**Fig. S10**	ORTEP view of MFA-DMAP-H_2O (1:1:1).	S11
**Fig. S11**	ORTEP representationof the MFA-DMAP (2:1).	S12
**Fig. S12**	Dihedral angles between phenyl rings in (a) TFA and (b) MFA of their DMF solvates.	S12
**Fig. S13**	PXRD patterns of TFA multicomponent solids and a polymorph.	S13
**Fig. S14**	PXRD patterns of MFA multicomponent solids.	S14
**Fig. S15**	DSC profiles of the TFA and MFA cocrystal/salt.	S15
**Fig. S16**	TGA profiles of the TFA and MFA cocrystal/salt.	S15

Table S1 Crystallographic data and structure refinement parameters of TFA multicomponent solids and a polymorph (TFA form-VI).

	TFA-DMAP (1:1)	TFA-DMAP-H_2O (1:1:1)	TFA-DMAP (2:1)	TFA form-VI	TFA-DMF
Formula	$C_{21}H_{22}ClN_3O_2$	$C_{21}H_{24}Cl_1N_3O_3$	$C_{35}H_{34}Cl_2N_4O_4$	$C_{14}H_{12}Cl_1N_1O_2$	$C_{17}H_{19}Cl_1N_2O_3$
Crystal system	triclinic	Triclinic	triclinic	triclinic	triclinic
Space group	$P\bar{1}$	$P\bar{1}$	$P\bar{1}$	$P\bar{1}$	$P\bar{1}$
a [Å]	7.9299(5)	7.7631(6)	10.8864(7)	6.7049(3)	10.4803(6)
b [Å]	9.3219(4)	8.0250(6)	12.2705(7)	7.2778(3)	11.8423(9)
c [Å]	13.5862(9)	16.2297(12)	13.7811(10)	14.1630(6)	13.3309(8)
α [^{0}]	87.768(5)	101.784(6)	106.966(6)	77.167(4)	94.335(6)
β [^{0}]	76.928(5)	98.374(6)	105.782(6)	79.908(3)	95.883(5)
γ [^{0}]	76.025(5)	90.687(6)	103.324(5)	65.487(4)	102.871(6)
V [Å ^{3}]	949.20(10)	978.32(13)	1595.53(19)	610.42(5)	1596.16(18)
Z	2	2	2	2	4
λ [Å]	0.71073	1.54184	0.71073	0.71073	0.71073
ρ_{calcd} [gcm^{-3}]	1.343	1.364	1.344	1.424	1.393
F[000]	404	424	676	272	704
μ [mm^{-1}]	0.223	1.957	0.249	0.305	0.256
θ [^{0}]	2.239- 26.278	2.81 – 66.16	3.914 - 26.35	2.96 - 25.03	1.77 - 26.37
Index ranges	$-9 \le h \le 9$<br>$-11 \le k \le 11$<br>$-16 \le l \le 15$	$-9 \le h \le 9$<br>$-9 \le k \le 8$<br>$-16 \le l \le 19$	$-12 \le h \le 12$<br>$-14 \le k \le 14$<br>$-16 \le l \le 16$	$-7 \le h \le 7$<br>$-8 \le k \le 8$<br>$-16 \le l \le 16$	$-13 \le h \le 12$<br>$-14 \le k \le 14$<br>$-16 \le l \le 16$
T [K]	290.80(10)	100	293(2)	100.00(10)	100.00(10)
R1	0.0567	0.0495	0.0755	0.0792	0.0527

*wR*2	0.1406	0.1406	0.1831	0.2157	0.1072
R_{merge}	0.0783	0.0583	0.1060	0.0842	0.0786
Parameters	339	265	582	168	423
GOF	1.054	1.047	1.058	1.060	1.042
Reflns total	6616	6172	8477	11414	15902
Unique reflns	3841	3366	5577	2133	6498
Obsd reflns	2799	2829	3784	1931	4756
CCDC	1532473	1532474	1532475	1532476	1532477

Table S2 Crystallographic data and structure refinement parameters of MFA multicomponent solids.

	MFA-DMAP (1:1)	MFA-DMAP-H_2O (1:1:1)	MFA-DMAP (2:1)
Formula	$C_{22}H_{25}N_3O_2$	$C_{23}H_{27}N_2O_3$	$C_{37}H_{40}N_4O_4$
Crystal system	Triclinic	Triclinic	triclinic
Space group	$P\bar{1}$	$P\bar{1}$	$P\bar{1}$
a [Å]	7.7575(8)	7.7248(3)	10.7678(8)
b [Å]	9.4727(7)	8.0592(3)	11.9673(10)
c [Å]	13.3076(13)	16.2531(7)	13.7860(11)
α [^{0}]	87.515(7)	101.711(4)	106.152(7)
β [^{0}]	78.596(8)	98.743(4)	105.842(7)
γ [^{0}]	74.174(8)	90.160(3)	103.490(7)
V [Å ^{3}]	922.20(15)	978.68(7)	1546.9(2)

Z	2	2	2
λ [Å]	0.71073	0.71073	1.54184
ρ_{calcd} [gcm^{-3}]	1.309	1.294	1.298
F[000]	388	408	644
μ [mm^{-1}]	0.085	0.087	0.680
θ [^{0}]	2.70- 26.32	2.56 – 28.08	3.56 - 50.80
Index ranges	-6≤ h ≤ 9<br>-9≤ k ≤ 11<br>-14≤ l ≤ 16	-9≤ h ≤9<br>-10≤ k ≤10<br>-20≤ l ≤20	-10≤ h ≤10<br>-10≤ k ≤12<br>-13≤ l ≤13
T [K]	100.02(10)	100.00 (10)	100.01(10)
R1	0.0621	0.0379	0.0637
wR2	0.1269	0.0937	0.1701
R_{merge}	0.0779	0.0418	0.0807
Parameters	341	266	525
GOF	1.104	1.035	1.036
Reflns total	5455	20122	4616
Unique reflns	3361	3976	3203
Obsd reflns	2670	3605	2494
CCDC	1532471	1532470	1532472

Table S3 Selected torsion angle (***τ***), the dihedral angle between planes of phenyl rings (***β***), of the TFA, MFA and their multicomponent solids, as well as the angle between the acid containing aromatic ring of fenamate and the DMAP pyridyl ring (**θ**).

	τ **(°)**	***β*** **(°)**	**θ (°)**
TFA form-I	−75	61.76	….
MFA form-I	−120.24	62.39	….
TFA-DMAP (1:1)	74.29; −72.62	88.50	81.89
MFA-DMAP (1:1)	73.71; −69.27	86.11	80.08
TFA-DMAP (1:1:1)	74.27	67.84	72.52
MFA-DMAP (1:1:1)	74.29	69.41	74.58
TFA-DMAP (2:1)	82.06; −70.54<br>−86.92; 74.68	81.40<br>78.43	87.00
MFA-DMAP (2:1)	91.68; −75.14<br>−90.51; 71.69	80.70<br>78.82	89.61

Table S4

1 (a) Geometrical parameters of hydrogen bonds observed in TFA-DMAP (1:1) crystal structure.

**D – H ··· A**	**D − H (Å)**	**H···A (Å)**	**D···A (Å)**	**∠D − H···A (°)**
N3 – H3A ··· O1	0.92(2)	1.67 (2)	2.578(3)	171(2)
C18 – H18 ··· O2	0.95	2.51	3.418(3)	161
C20 – H20B ··· O2	0.98	2.51	3.440(3)	158
C21 – H21B ··· O2	0.98	2.52	3.462(3)	162

1 (b) Geometrical parameters of hydrogen bonds observed in MFA-DMAP (1:1) crystal structure.

**D – H ··· A**	**D − H (Å)**	**H···A (Å)**	**D···A (Å)**	**∠D − H···A (°)**
N3 – H3 ··· O1	0.92(2)	1.65 (2)	2.570(2)	172(2)
C18 – H18 ··· O2	0.93	2.51	3.416(3)	163
C21 – H21B ··· O2	0.96	2.51	3.443(3)	163
C22 – H22B ··· O2	0.96	2.53	3.459(3)	162

2 (a) Geometrical parameters of hydrogen bonds observed in TFA-DMAP-H_2O (1:1:1) crystal structure.

**D – H ··· A**	**D − H (Å)**	**H···A (Å)**	**D···A (Å)**	**∠D − H···A (°)**
N3 – H3A ··· O1	0.86(2)	1.85(2)	2.703(2)	177(2)
O3 – H3B ··· O2	0.87	1.94	2.775(2)	160
O3 – H3C ··· O1	0.87	2.01	2.852(2)	164
C16 – H16 ··· O3	0.95	2.40	3.320(3)	164
C20 – H20B ··· O2	0.98	2.53	3.442(3)	155

2 (b) Geometrical parameters of hydrogen bonds observed in MFA-DMAP-H_2O (1:1:1) crystal structure.

**D – H ··· A**	**D − H (Å)**	**H···A (Å)**	**D···A (Å)**	**∠D − H···A (°)**
N3 – H3A··· O1	0.917(16)	1.786(16)	2.697(15)	173.2(15)
O3 – H3B ··· O2	0.85	1.95	2.772(14)	164
O3 – H3C··· O1	0.85	2.03	2.848(13)	160
C17 – H17 ··· O3	0.93	2.42	3.333(16)	166
C21 – H21B··· O2	0.96	2.54	3.433(17)	155

3 (a) Geometrical parameters of hydrogen bonds observed in TFA-DMAP (2:1) crystal structure.

**D – H ··· A**	**D − H (Å)**	**H···A (Å)**	**D···A (Å)**	**∠D − H···A (°)**
O3 – H3 ··· O2	0.84(4)	1.72(4)	2.543(4)	170(5)
N4 – H4A ··· O1	0.89(4)	1.82(4)	2.667(5)	158(4)
C34 – H34B ··· O4	0.96	2.59	3.515(5)	162
C35 – H35B ··· O4	0.96	2.43	3.375(5)	170

3 (b) Geometrical parameters of hydrogen bonds observed in MFA-DMAP (2:1) crystal structure

**D – H ··· A**	**D − H (Å)**	**H···A (Å)**	**D···A (Å)**	**∠D − H···A (°)**
O3 – H3 ··· O2	0.85(4)	1.70(4)	2.546(3)	175(3)
N4 – H4A ··· O1	0.92(4)	1.75(4)	2.645(4)	166(3)
C3 – H3A ··· N3	0.95	2.61	3.526(5)	163
C36 – H36B ··· O4	0.98	2.40	3.364(5)	168
C37 – H37B ··· O1	0.98	2.52	3.448(5)	158

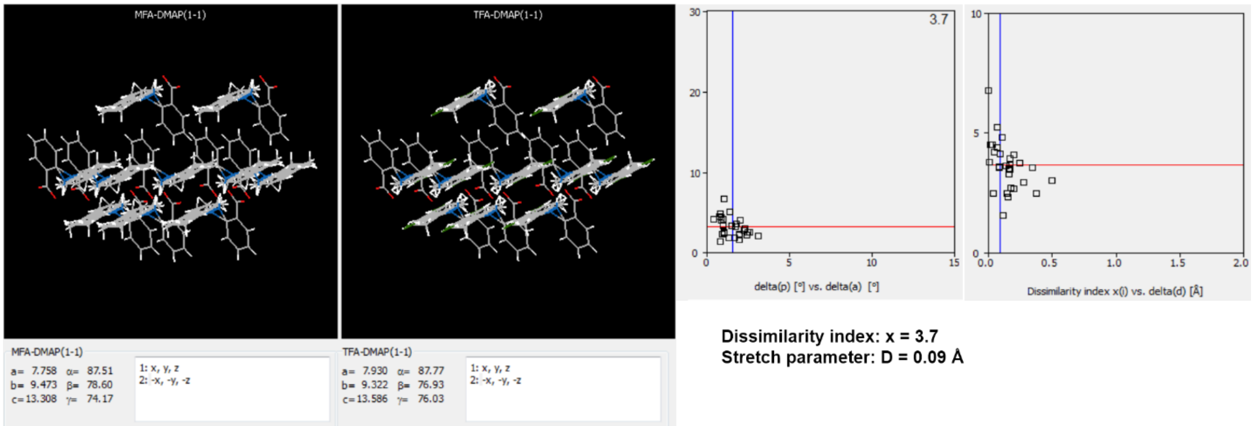

Fig. S1 3D supramolecular constructs between MFA-DMAP (1:1) and TFA-DMAP (1:1) deduced from XPac analysis.

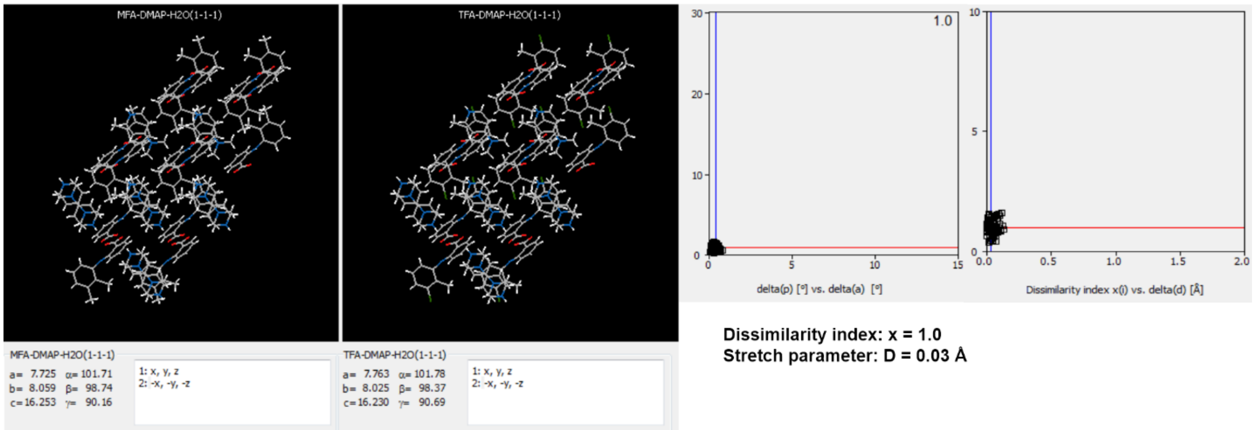

Fig. S2 3D supramolecular constructs between MFA-DMAP-H_2O (1:1:1) and TFA-DMAP-H_2O (1:1:1) deduced from XPac analysis.

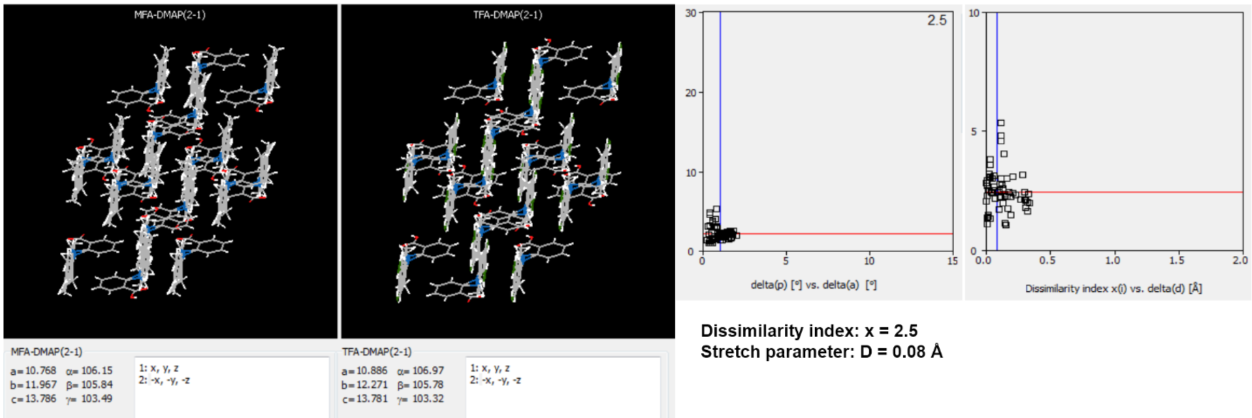

Fig. S3 3D supramolecular constructs between MFA-DMAP (2:1) and TFA-DMAP (2:1) deduced from XPac analysis.

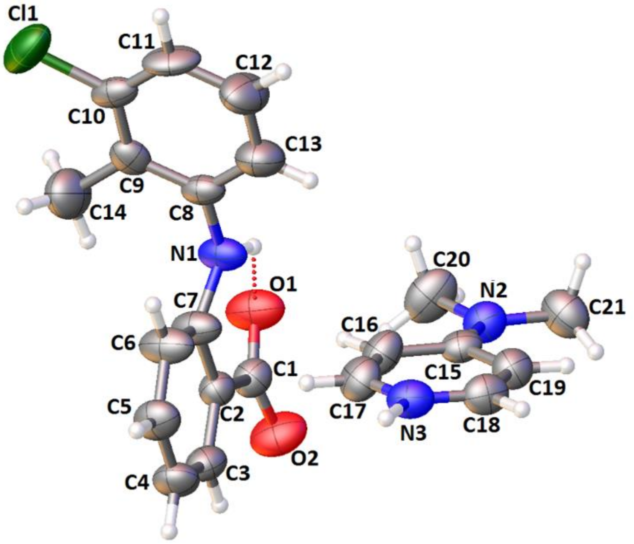

Fig. S4 ORTEP representation of TFA-DMAP (1:1) salt, where the displacement ellipsoids were drawn at 50% probability level.

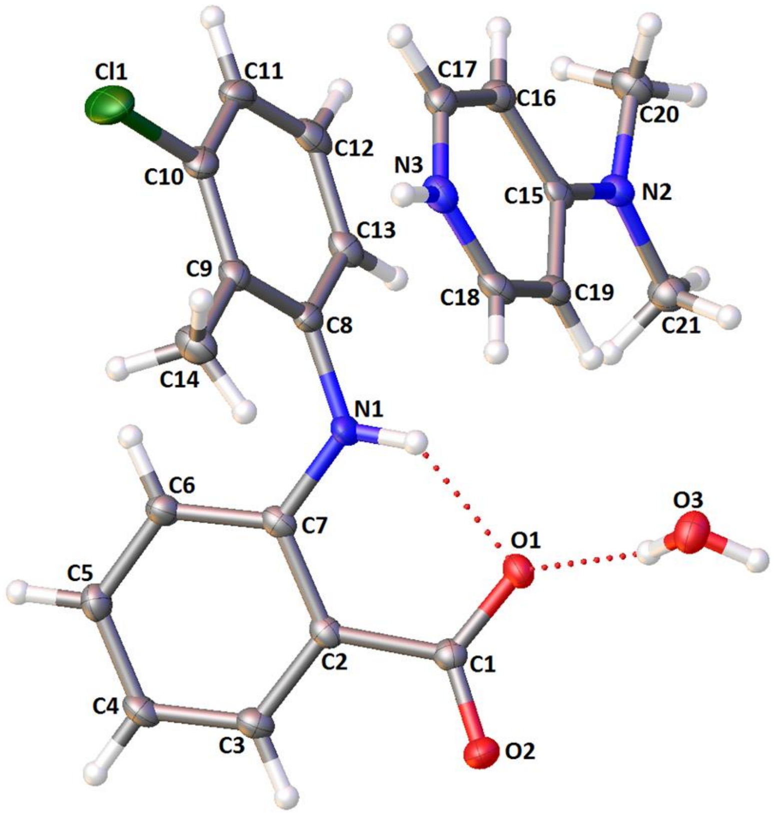

Fig. S5 ORTEP representationof the TFA-DMAP-H_2O (1:1:1) with 50% probability of displacement ellipsoids.

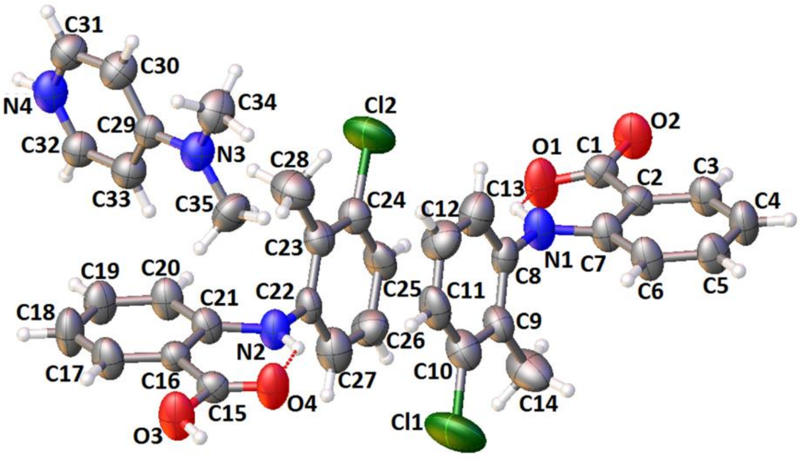

Fig. S6 ORTEP view of TFA-DMAP (2:1), here the displacement ellipsoids were drawn at 50% probability level.

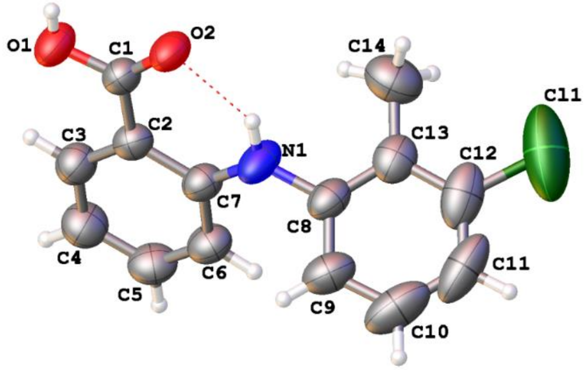

Fig. S7 ORTEP view of TFA, here the displacement ellipsoids were drawn at 50% probability level.

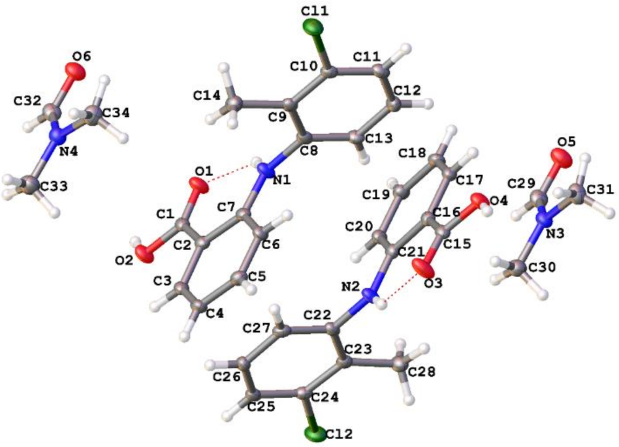

Fig. S8 ORTEP view of TFA-DMF solvate, here the displacement ellipsoids were drawn at 50% probability level.

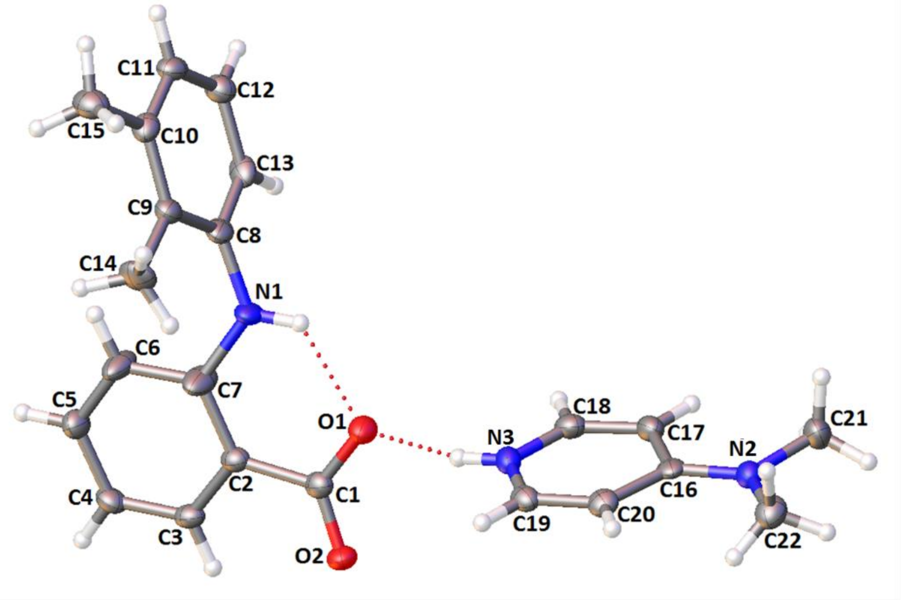

Fig. S9 ORTEP representation of MFA-DMAP (1:1) where the displacement ellipsoids were drawn at 50% probability level.

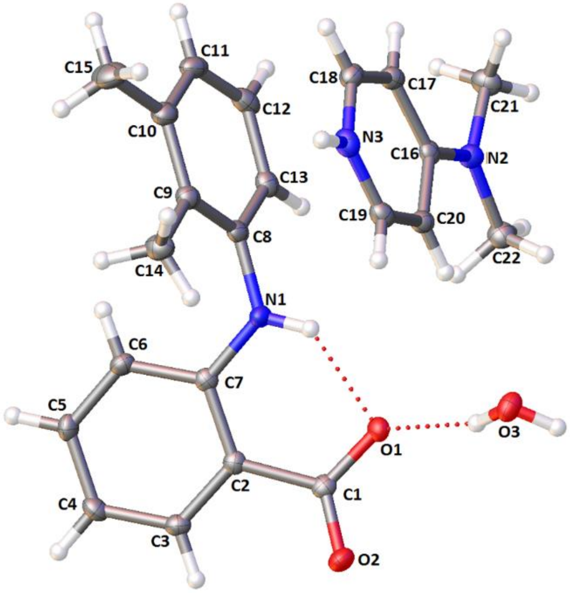

Fig. S10 ORTEP representation of the MFA-DMAP-H_2O (1:1:1) with 50% probability of displacement ellipsoids.

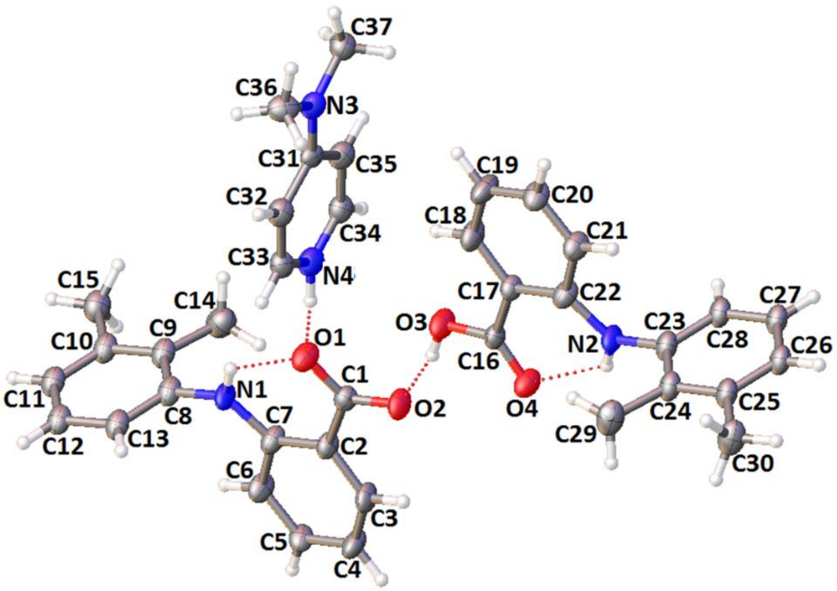

Fig. S11 ORTEP view of MFA-DMAP (2:1), here the displacement ellipsoids were drawn at 50% probability level.

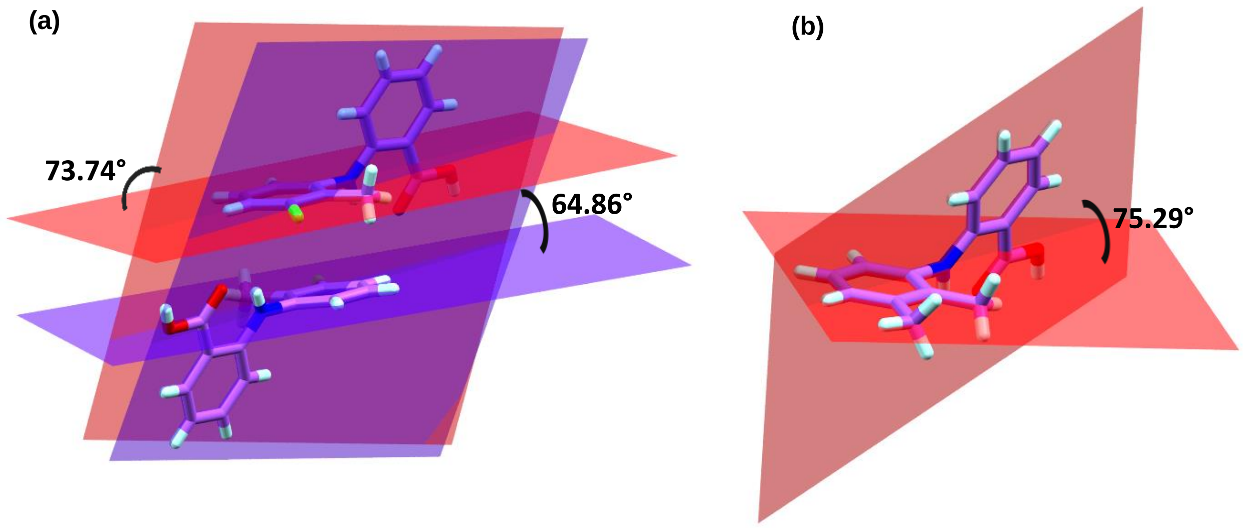

Fig. S12 Dihedral angles between phenyl rings in (a) TFA and (b) MFA of TFA-DMF and MFA-DMF solvates, respectively.

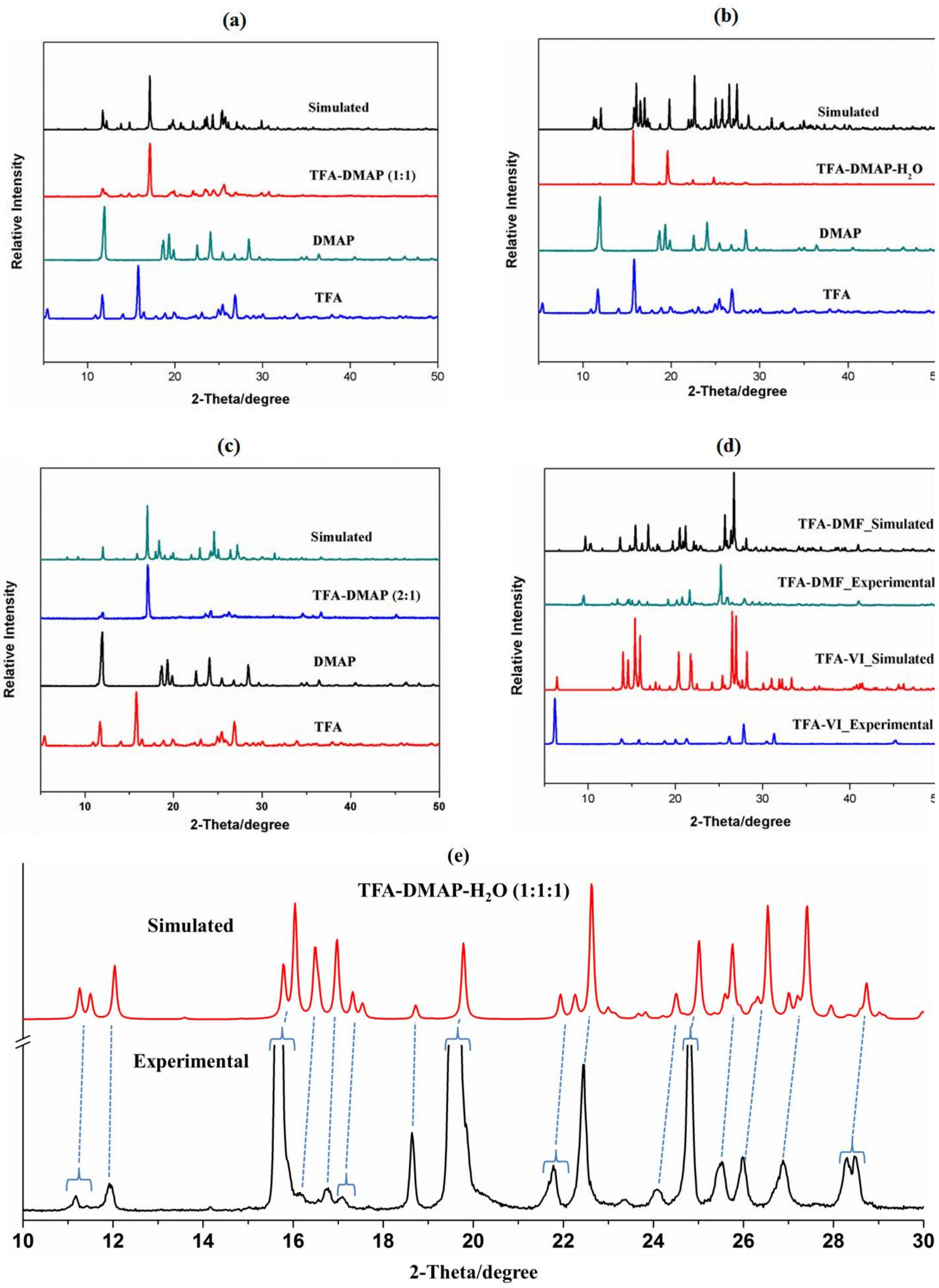
(a)
Simulated
TFA-DMAP (1:1)
DMAP
TFA
Relative Intensity
2-Theta/degree
(b)
Simulated
TFA-DMAP-H2O
DMAP
TFA
Relative Intensity
2-Theta/degree
(c)
Simulated
TFA-DMAP (2:1)
DMAP
TFA
Relative Intensity
2-Theta/degree
(d)
TFA-DMF_Simulated
TFA-DMF_Experimental
TFA-VI_Simulated
TFA-VI_Experimental
Relative Intensity
2-Theta/degree
(e)
TFA-DMAP-H2O (1:1:1)
Simulated
Experimental
2-Theta/degree

Fig. S13 PXRD patterns of (a) TFA-DMAP (1:1) (b) TFA-DMAP-H_2O (1:1:1) (c) TFA-DMAP (2:1) and (d) TFA form-VI and TFA-DMF as-synthesized and simulated from the single-crystal data. (e) The amplified experimental and simulated PXRD patterns of TFA-DMAP-H_2O are shown for clarity.

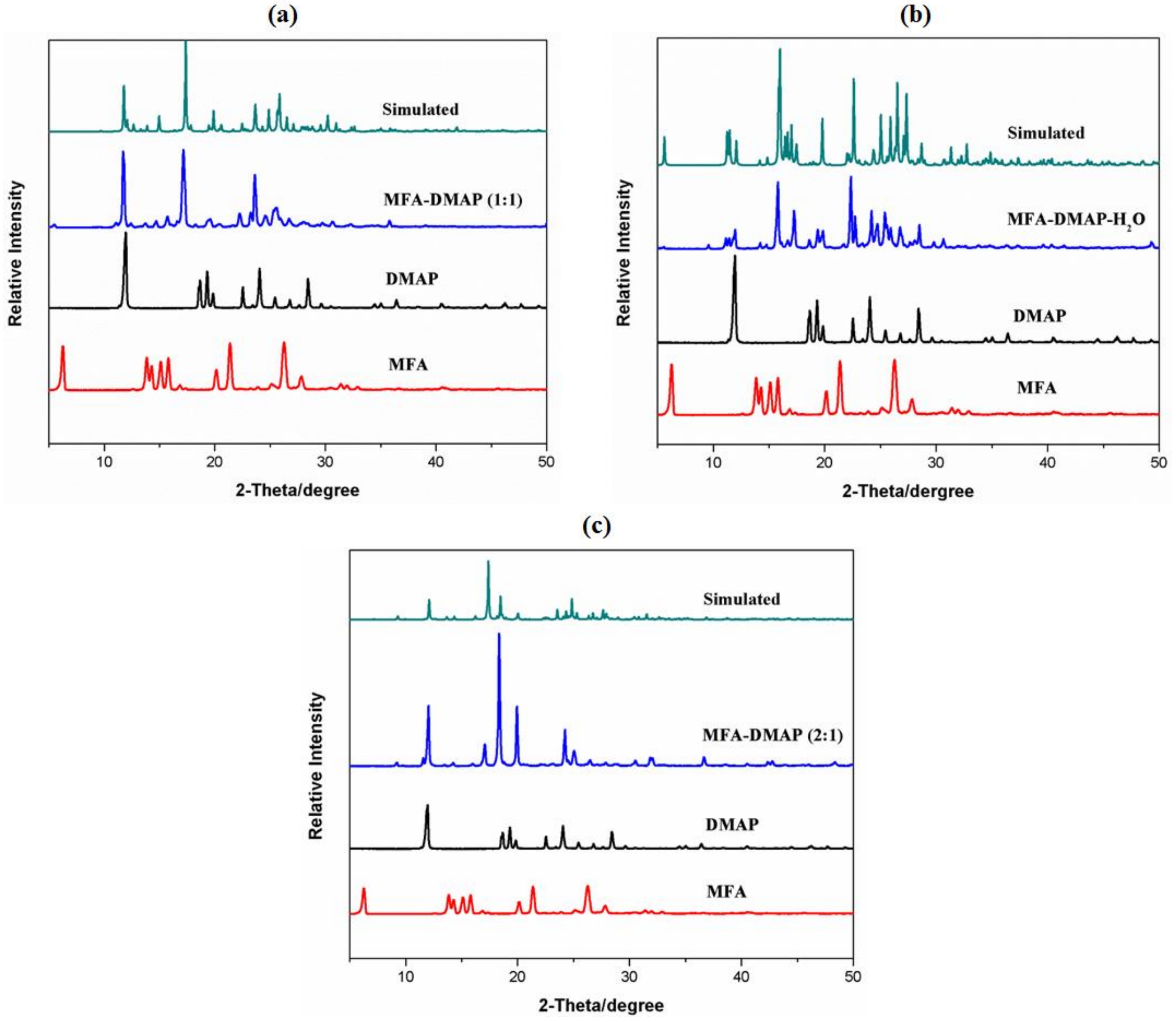

Fig. S14 PXRD patterns of (a) MFA-DMAP (1:1) (b) MFA-DMAP-H_2O (1:1:1) and (c) MFA-DMAP (2:1) as-synthesized and simulated from the single-crystal data.

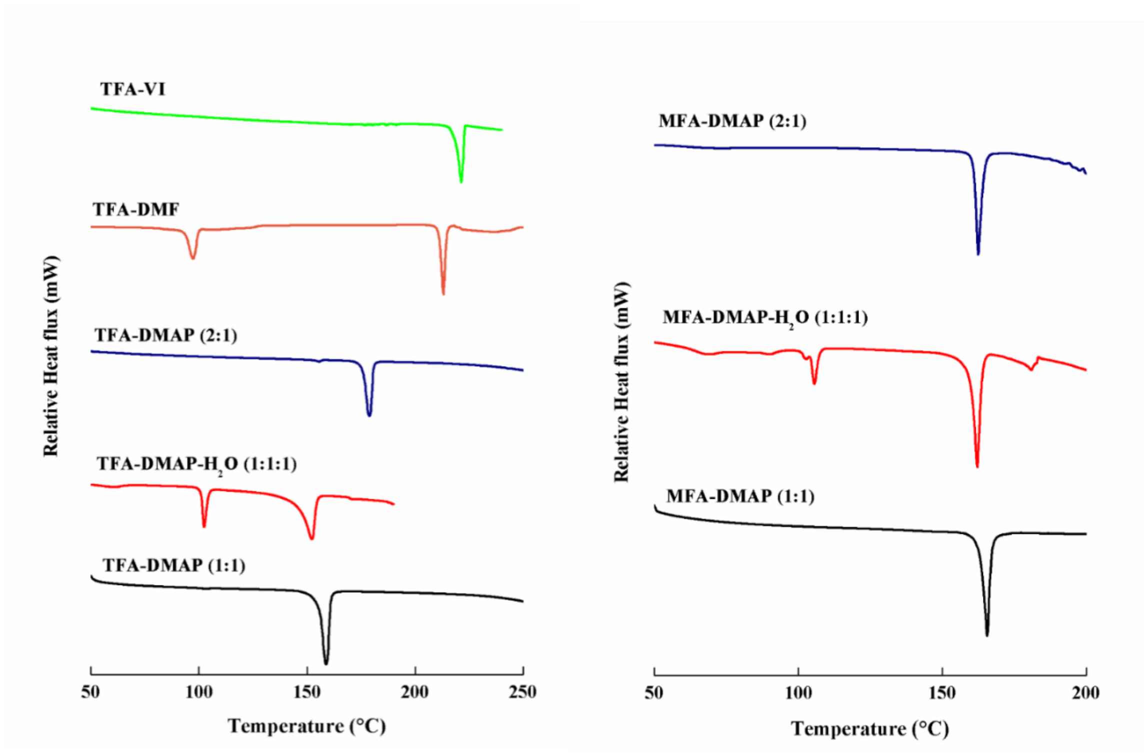

Fig. S15 DSC profiles of (a) TFA single and multicomponent solids and (b) MFA multicomponent solids.

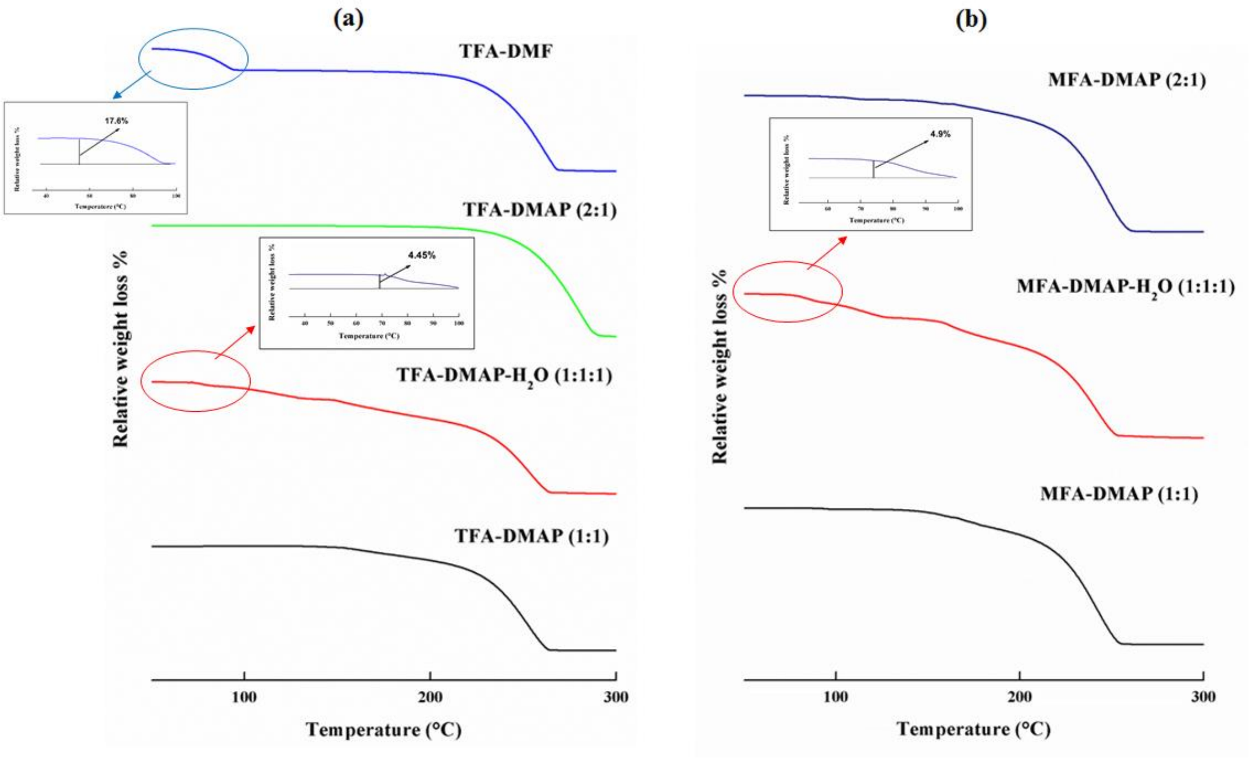

Fig. S16 Thermogravimetric profiles of the (a) TFA and (b) MFA multicomponent solids.
